# Neuroscience: large-scale evidence for perceptual entrainment to auditory rhythms

**DOI:** 10.1038/s44271-025-00315-5

**Published:** 2025-09-12

**Authors:** David Pascucci

**Affiliations:** 1 Psychophysics and Neural Dynamics Lab, The Radiology Department, Lausanne University Hospital and University of Lausanne, Lausanne, Switzerland; 2 The Sense Innovation and Research Center, Lausanne, Switzerland

**Keywords:** Cognitive neuroscience

## Abstract

A large multi-lab replication study confirms that rhythmic sounds can entrain perceptual performance, while revealing substantial inter-individual variability.

**Figure:**
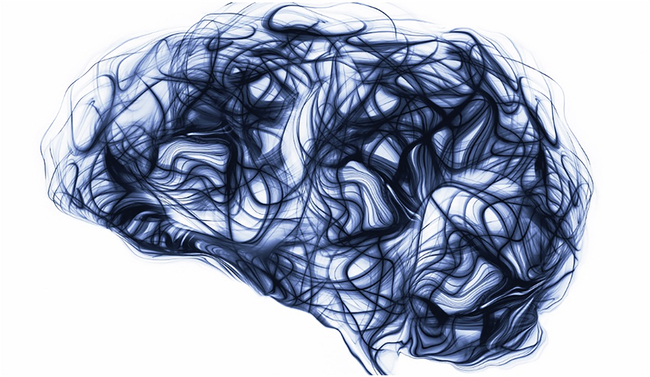
© SEAN GLADWELL/Moment/Gettyimages

What do music, sport, and the fascinating flashing of fireflies have in common? Rhythms and synchrony. They pervade both nature and human activity, often emerging spontaneously through entrainment—a process where two systems fall into step with one another. A key question is whether rhythmic sensory input can entrain the brain’s intrinsic activity, influencing perception and cognition.

Molly Henry at the Max Planck Institute for Empirical Aesthetics and an extensive group of collaborators, as part of a large multi-lab consortium spanning over 30 research institutions, addressed this question^1^ through a preregistered replication of a landmark study^2^. One of the key predictions of entrainment research in neuroscience is that the alignment between rhythmic stimulation and neural activity can persist even after the rhythm disappears, leading to rhythmic fluctuations in subsequent perceptual performance. With 5 participants, the original study confirmed the prediction—specifically, that the ability to detect target stimuli fluctuates at the rhythm of a preceding sound even after the sound ceases to be rhythmic.

This large-scale replication confirmed and extended those findings. With a much larger sample size (n = 149) and broader analyses, the team reproduced the key behavioral effect: detection accuracy systematically fluctuated in synchrony with the waxing and waning of a prior rhythmic sound. The study also revealed substantial variability in the strength of the entrainment effect, not only across individual participants but also among the participating labs. This variability was not fully explained by demographic factors (e.g., age) or by self-reported measures such as task engagement, communication ability, or musical sophistication. Characterizing this variability is a key contribution of the study. As the authors note:

“We had individually tried extensions of the original paradigm in our labs but were unable to replicate what we saw as the core finding. These initial failures to replicate got us thinking that we should do a more careful replication attempt to understand how robust the original finding was.”

Thus, had any one lab run this study alone, they might have arrived at conflicting conclusions.

While the replication reinforces the idea that rhythmic stimuli can entrain perception, important questions remain—such as whether the observed effects come from the auditory or attention system. Nevertheless, this large-scale replication sets a good example for the importance of testing the reliability of psychological phenomena. Practically, understanding the robustness of these effects and the nature of inter-individual variability can lay the groundwork for more nuanced, evidence-based approaches to personalized interventions.
